# Effects of Mixed Controlled Release Nitrogen Fertilizer with Rice Straw Biochar on Rice Yield and Nitrogen Balance in Northeast China

**DOI:** 10.1038/s41598-020-66300-6

**Published:** 2020-06-11

**Authors:** Yu Zheng, Xiaori Han, Yuying Li, Shuangquan Liu, Jinghong Ji, Yuxin Tong

**Affiliations:** 1 College of Land and Environment, Shenyang Agricultural University, Shengyang, 110866 China; 2 Institute of Soil Fertilizer and Environmental Resources, Heilongjiang Academy of Agricultural Sciences, Harbin, 150086 China

**Keywords:** Agroecology, Environmental impact

## Abstract

A 3-year fixed site experiment was carried out on a Planosol in Northeast China to study the effects of biochar and controlled-release nitrogen fertilizer on rice yield, nitrogen-use efficiency, residual nitrogen, and nitrogen balance in soil-crop system. Five treatments were established: control (CK), bare urea (BU), controlled-release urea (CRU), 50% BU + 50% CRU (MBC), and 50% BU + 50% CRU + biochar (MBCB) treatments. The results showed that, compared with the BU treatment, the yield, N-use efficiency (NUE) and N agronomic efficiency (NAE) of the CRU treatment increased by 12.2%, 33.9% and 4.3 kg kg^−1^, respectively; while the soil residual N and N surplus at harvest decreased by 11.6% and 10.7%, respectively. Compared with the MBC treatment, the yield, NUE and NAE of the MBCB treatment increased by 10.2%, 16.5% and 4.0 kg kg^−1^, respectively; while the soil residual N and N surplus at harvest decreased by 10.8% and 12.3%, respectively. Therefore, mixed application of bare urea, controlled-release urea and biochar was effective for obtaining high rice yield, and high fertilization efficiency as well as for sustainable agricultural development in Northeast China.

## Introduction

Controlled-release fertilizers are typically coated with many different types of materials, such as paraffin, resin, natural rubber, polychlorovinyl and polylactic acid^1^, etc. These coated fertilizers are mainly nitrogenous fertilizers, and their function is to delay the absorption and utilization of fertilizer by their target plants; as such, the absorption and utilization time of their target plants is significantly longer than that of ordinary nitrogenous fertilizers^2,3^. The most widely used controlled-release fertilizer is the controlled-release urea (CRU), which is usually resin coated. CRU is more effective than bare urea (BU) at increasing crop yield and nitrogen fertilizer-use efficiency^4^. There have been many reports about the advantages of nutrient release and crop absorption steps associated with controlled-release nitrogen fertilizer in rice with mixed application techniques^5–7^. Single mixed application of controlled-release nitrogen fertilizer and ordinary urea can improve the proportion of dry matter and nitrogen accumulation in rice^8^. The use of CRU is a specific practice to synchronize crop nitrogen demand, which could minimize early-season N availability when crop uptake is slow, thereby reducing the loss potential and saving labour by a one-off application^9^.

Biochar has highly porous structure, large surface area, and high ion-exchange capacity and can impact a number of processes in the soil N cycle associated with enhanced soil fertility^10,11^. It has been reported that biochar application increases soil nutrient retention capacity and nitrogen-use efficiency (NUE), improves soil fertility and reduces of NO_3_^–^-N leaching in soils^12–15^.

Rice is the staple food for more than 65% of the population and the perennial planting area is approximately 30.2 million hectares in China^16^. Heilongjiang Province is one of the most important commercial grain production bases in China, with 12.3 million hectares of cultivated land; the rice planting area accounts for 26% of the province’s grain crop planting area and 10.4% of the country’s rice planting area^17^. Mixed application of CRU and BU have been widely used in current production practices in China in recent decades; farmers mainly use the fertilization technique of one basal and two topdressing application for rice planting, but this usually results in fast nutrient release, low fertilizer-use rate and relatively low yield^18^. To achieve high crop yield, new methods, such as biochar application, have been introduced. Although how biochar amendment affects crop yield and nitrogen balance has been reported extensively^10–15^, little information is available on how mixed application of biochar with CRU and BU affect crop yield, the nitrogen use rate, residual nitrogen and nitrogen balance in soil-crop systems.

Therefore, the methodology of a fixed-site experiment under equal nutrient contents of NPK was adopted. The objectives of this study were to investigate the effects of mixture of biochar with controlled-release nitrogen fertilizer and ordinary nitrogen fertilizer on rice yield, nitrogen-use efficiency, and nitrogen balance after a three years application. This study could provide a theoretical basis for the mixed application of biochar with nitrogen fertilizers in current cropland management systems, especially for local farmers in the cold region of Northeast China.

## Results

### Effects of biochar and controlled-release nitrogen fertilizer on rice yield

**Figure 1:**
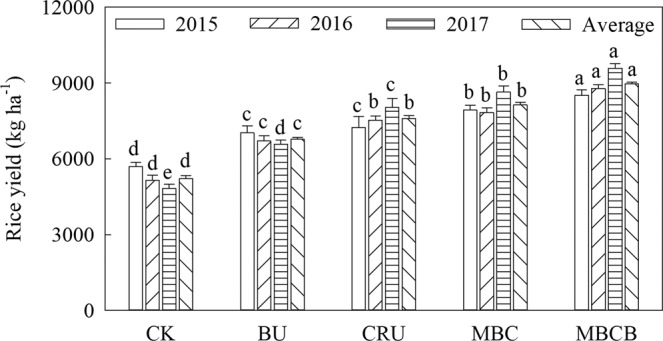
Rice yield of the different treatments. Data here are mean ± standard deviation, *n* = 3. The same lowercase letters indicated no significant differences among different fertilization treatments in the same year according to LSD test (p < 0.05). The same in Figs. 2 to 7.

The different nitrogen fertilizer management practices had a significant effect on rice yield (Fig. 1, p < 0.05). Compared with the CK treatment, the yields of the treatments of BU, CRU, MBC and MBCB significantly increased by 29.7%, 45.5%, 55.7% and 71.5%, respectively. In addition, compared with that in the BU treatment, the yield in the CRU treatment increased by 12.2%. The yield in the MBCB treatment increased by 10.2% as compared to that in the MBC treatment.

### Effects of biochar and controlled-release nitrogen fertilizer on the N uptake of the rice

**Figure 2:**
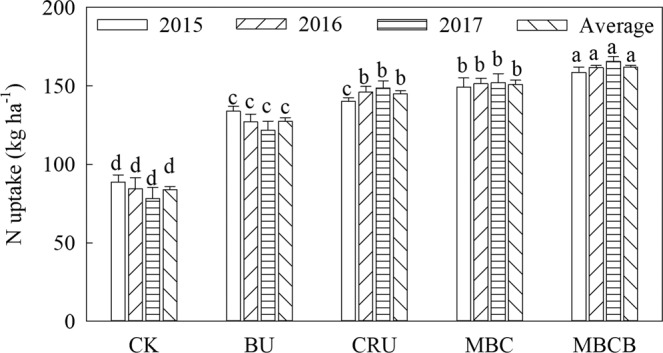
N uptake of different treatments of rice.

The results showed that the N uptake and N utilization of rice in the CRU treatment were higher than that in the BU treatment (Fig. 2, p < 0.05). The nitrogen uptake for the treatments of BU, CRU, MBC and MBCB significantly increased by 28.3%, 73.0%, 80.0% and 91.1% over that of the CK, respectively. In addition, compared with the BU treatment, the N uptake in CRU treatment increased by 13.7%; the N uptake in the MBC treatment increased by 4.1% as compared to that in the CRU treatment; and the N uptake in MBCB treatment increased by 7.4% compared to that in the MBC treatment.

### Effects of biochar and controlled-release nitrogen fertilizer on N-use efficiency

**Figure 3:**
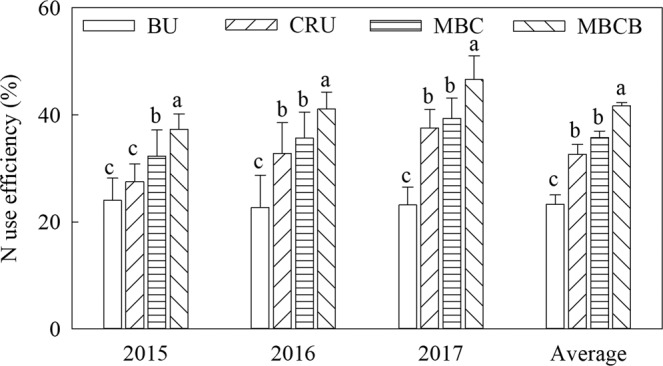
N fertilizer-use efficiency of the different treatments.

**Figure 4:**
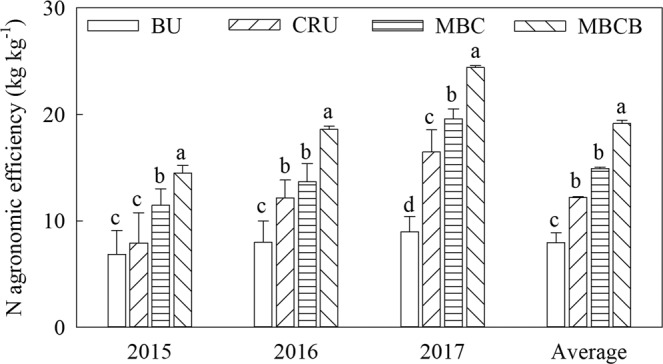
Agronomic efficiency of N in the different treatments.

The nitrogen-use efficiency (NUE) was greatly influenced by the different nitrogen fertilizer treatments (Fig. 3, p < 0.05). Compared with the BU treatment, the NUE in the CRU treatment increased by 33.9%; the NUE in the MBC treatment increased by 9.8% compared to that in the CRU treatment; and the NUE in the MBCB treatment increased by 16.5% compared to that in the MBC treatment. The nitrogen agronomic efficiency (NAE) was significantly affected by the different nitrogen fertilizer treatments (Fig. 4, p < 0.05). Compared with the BU treatment, the NAE of the CRU treatment increased by 4.3 kg kg^−1^; the NAE of the MBC treatment increased by 2.7 kg kg^−1^ compared to that of the CRU treatment; the NAE of MBCB treatment increased 4.0 kg kg^−1^ compared to that of the MBC treatment.

### Effects of biochar and controlled-release nitrogen fertilizer on the inorganic N content in the soil profile

**Figure 5:**
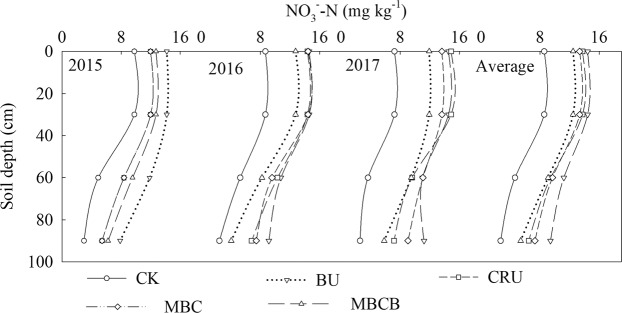
NO_3_^-^-N content in the soil profile during 2015–2017.

**Figure 6:**
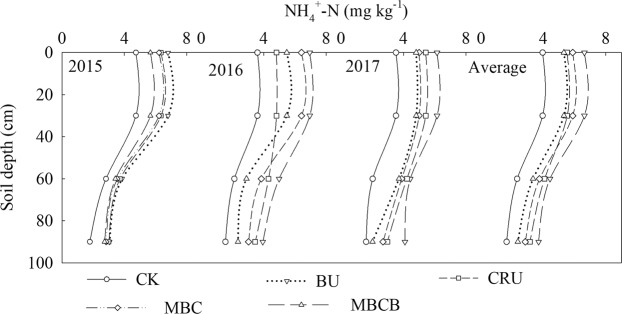
NH_4_^+^-N content in the soil profile during 2015–2017.

The results showed that the contents of NO_3_^–^-N and NH_4_^+^-N in the soil profile (0–90 cm) in response to N application were significantly higher than those in the CK treatment (Fig. 5, p < 0.05). Compared with the CK treatment, the content of NO_3_^–^-N increased by 130.9%, 113.6% and 163.8% in the 0–30 cm, 30–60 cm and 60–90 cm layers, respectively. Besides, the NO_3_^–^-N of the CRU treatment decreased by 6.2%, 17.5% and 27.6% in the 0–30 cm, 30–60 cm and 60–90 cm layers, respectively, as compared to that in the BU treatment; the NO_3_^–^-N of the MBCB treatment decreased by 11.9%, 13.0% and 23.3% in the 0–30 cm, 30–60 cm and 60–90 cm layers, respectively, as compared to that in the MBC treatment. The trend of the variation in the content of NH_4_^+^-N in the soil profile was the same trend as that of NO_3_^–^-N (Fig. 6, p < 0.05). Compared with the CK treatment, the average content of NH_4_^+^-N in the four N application treatments increased by 45.6%, 59.7% and 68.2% in the 0–30 cm, 30–60 cm and 60–90 cm layers, respectively. In addition, the NH_4_^+^-N of the CRU treatment decreased by 16.6%, 13.5% and 19.5% in 0–30 cm, 30–60 cm and 60–90 cm layers, respectively, as compared with that of the BU treatment; the NH_4_^+^-N of the MBCB treatment decreased by 9.7%, 15.4% and 20.1% in the three layers, respectively, as compared with that of the MBC treatment.

### Effects of biochar and controlled-release nitrogen fertilizer on inorganic nitrogen accumulation in the soil profile

**Figure 7:**
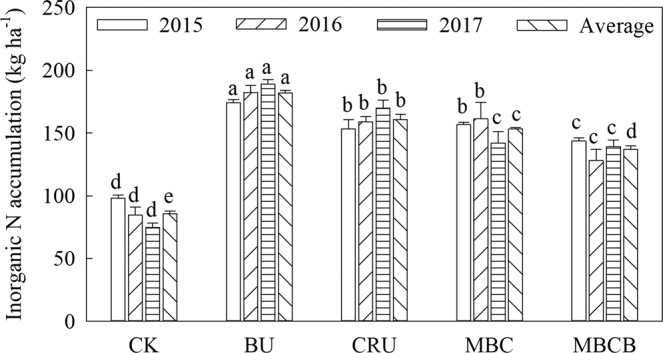
Inorganic N accumulation in the 0–90 cm soil profile.

**Figure 8:**
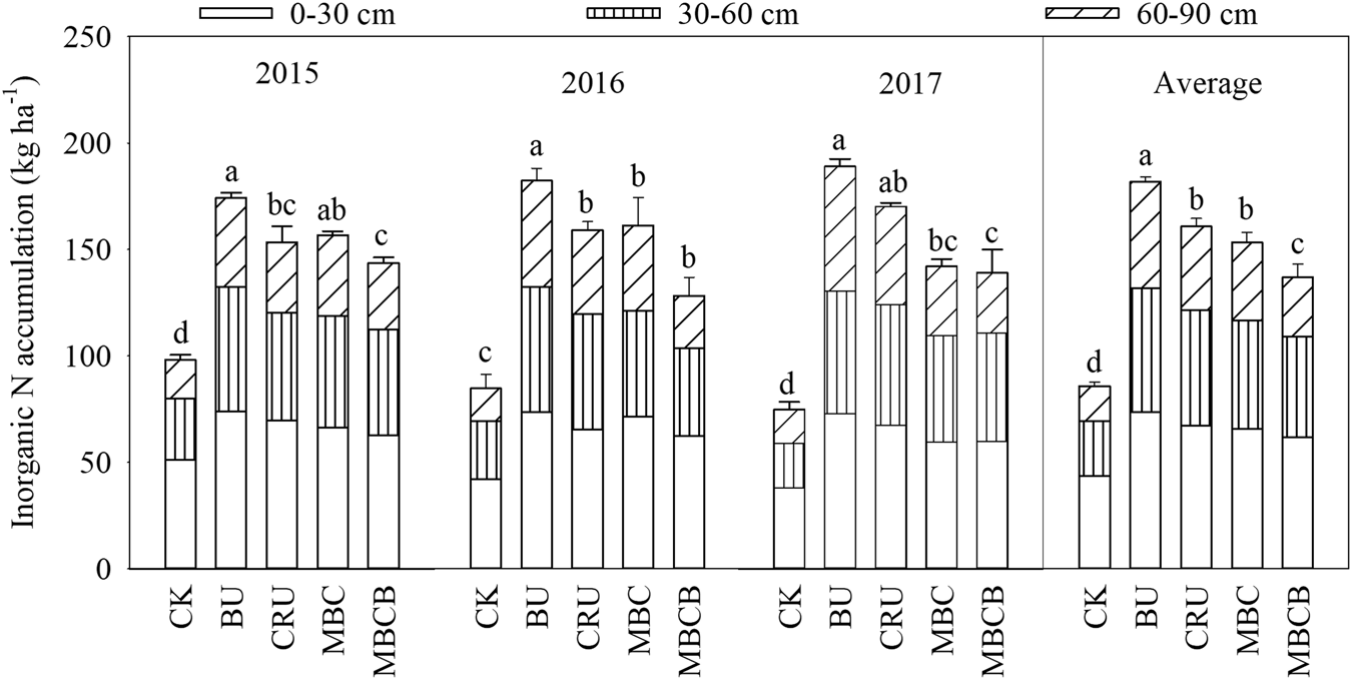
Inorganic N distribution in the 0–90 cm soil profile.

Soil inorganic nitrogen (SIN) mainly refers to NO_3_^-^-N and NH_4_^+^-N. The results showed that the total SIN accumulation ranked as follows: BU > CRU > MBC > MBCB > CK (Fig. 7, p < 0.05). Compared with the BU treatment, the average amount of SIN in the CRU treatment decreased by 11.6%; compared with the MBC treatment, the average amount of SIN in the MBCB treatment decreased by 10.8%. The average distribution of SIN in the soil profile was 44.4%, 32.4% and 23.3% in the 0–30 cm, 30–60 cm, and 60–90 cm layers for all the treatments, respectively (Fig. 8, p < 0.05). For the accumulation of SIN in the profile (0–90 cm) for all the treatments, NO_3_^-^-N and NH_4_^+^-N accounted for 69.7% and 30.2% on average, respectively. Compared with the BU treatment, the SIN accumulation of the CRU treatment decreased by 8.3%, 7.2% and 21.4% in the 0–30, 30–60 and 60–90 cm layers, respectively; compared with the MBC treatment, the SIN accumulation of the MBCB treatment decreased by 6.2%, 6.8% and 24.1% in the 0–30, 30–60 and 60–90 cm layers, respectively.

### Effects of biochar and controlled-release nitrogen fertilizer on the N balance in the soil-crop system

The controlled-release urea and biochar showed a significant effect on residual inorganic N and the apparent loss and surplus in the soil-crop system (Table 1, p < 0.05). Compared with the BU and MBC treatments, the residual SIN of the CRU and MBCB treatments on average decreased by 13.1% and 15.4%, respectively (p < 0.05). In addition, the apparent loss of N in the CRU and MBCB treatments on average decreased by 5.7% and 5.9%, respectively (p < 0.05). The inorganic N surplus decreased in the order of BU, CRU, MBC, MBCB and CK. The inorganic N surplus in the MBCB treatment decreased by an average of 12.3% compared to that in the MBC treatment; and the inorganic N surplus in the CRU treatment decreased by an average of 10.7% compared to that in the BU treatment.

**Table 1:** Nitrogen balance of different treatment for a three-year interval of 2015–2017.

Parameters	Ninitial	Nfertilizer	Nmineral	Nuptake	Nresidual	Napparentloss	Nsurplus
CK	111.4 ± 20.8b*	0.0 ± 0.0	44.2 ± 9.9	83.8 ± 5.2d	86.1 ± 11.7d	−14.3 ± 16.3b	71.8 ± 27.9d
BU	163.2 ± 26.5a	187.5 ± 0.0	44.2 ± 9.9	127.5 ± 6.0c	181.8 ± 7.4a	85.6 ± 15.7a	267.4 ± 20.9a
CRU	152.1 ± 16.7a	187.5 ± 0.0	44.2 ± 9.9	145.0 ± 4.3b	158.1 ± 4.3b	80.7 ± 14.8a	238.8 ± 13.1ab
MBC	159.1 ± 22.8a	187.5 ± 0.0	44.2 ± 9.9	150.9 ± 1.5b	161.5 ± 4.8b	78.4 ± 13.3a	239.9 ± 12.9ab
MBCB	140.8 ± 9.6ab	187.5 ± 0.0	44.2 ± 9.9	162.0 ± 3.5a	136.7 ± 7.9c	73.8 ± 21.5a	210.5 ± 19.5b

*Data here are mean ± SE, *n* = 3. The same lowercase letters refer to soil chemical properties were not significantly different among different fertilization treatments according to LSD test (*p* < 0.05).

Ninitial: residual NO_3_^¯^-N + NH_4_^+^-N (in 0–90 cm soil depth before sowing).

Nfertilizer: N fertilizer rate.

Nuptake: N uptake by above-ground parts at harvest.

Nresidual: accumulation of NO_3_^¯^-N + accumulation of NH_4_^+^-N(in 0–90 cm soil depth after harvest).

N mineralization (Nmin) = N uptake +N residual - N initial.

Napparent loss: Ninitial + Nfertilizer + Nmineral–(N uptake + N residual).

Nsurplus: Napparent loss + Nresidual.

## Discussion

### Effects of different fertilization management practices on rice yield

In the present study, the average rice yields of the CRU and MBC treatments were significantly greater than that of the BU treatment. This was consistent with the results of previous studies^19,20^, which showed that the controlled-release urea could significantly increase rice yield. For instance, a study found that CRU application with common urea at a ratio of 4:6 or 5:5 could increase rice yield by 8~34.2%^15,21^. This indicated that MBC (50% BU + 50% CRU) had a better effect on yield than did CRU or BU alone. In the present study, the rice yield of the MBCB treatment increased by 10.2% compared with that of the MBC treatment. This result was consistent with the other reports of yield increases of 9.5–13.7%^22,23^. This may be due to the acidity, poor physical and chemical properties of the tested soil. When biochar and NPK fertilizers were applied together, there was a strong synergistic effect on the increase in both grain and biomass yield^24^. However, biochar application alone had different effects on crop yield of different in biochar properties or soil properties, crop species and environmental conditions^25^. These results indicated that MBCB (50% BU + 50% CRU + biochar) had the greatest effect on rice yield. A possible reason was that the CRU and BC treatments reduced nitrogen loss, increased nitrogen-use efficiency and improved the soil physical and chemical properties^26,27^.

### Effects of different fertilization management practices on N uptake and N-use efficiency

The uptake of N by crops reflects the utilization capacity of the crop itself, and reflects the NUE. In the present study, CRU had a better effect on N uptake than did BU. This was consistent with previous reports, which noted that CRU could increase the N uptake and N-use efficiency of rice by 15.6% and 8.3%, respectively^19,20^. A possible reason was that CRU increased the crop N uptake from the fertilizer by the crops and reduced unidentified losses of applied N^28,29^. CRU can provide a continuous nitrogen supply for rice growth and development throughout the growth period^30^. In addition, MBCB also had a better effect on N uptake than did MBC. Previous studies also found that biochar used as compound fertilizer and combined with chemical nutrients could significantly increase both nitrogen-use efficiency and agronomic-use efficiency by 43.1%^7,31^. This may be because the adsorption function of biochar improves soil physical and chemical properties, making them more conducive to the growth of rice roots^32^.

The N requirement of crops is the most important factor influencing N-use efficiency^33^. In the present study, the NUE and NAE of the CRU treatment were significantly greater than those of the BU treatment. This is consistent with previous studies that reported that controlled-release nitrogen fertilizer mixed with bare urea could increase the apparent N-use efficiency by 20.0% compared with that of the BBF treatments at the same application rates^34^. This may be because CRU can increase N uptake by the plants and reduce N loss^35,36^. In addition, in the present study, the NUE and NAE of the MBCB treatment were significantly greater than those of the MBC treatment. Previous studies noted that biochar application alone led to an increase of 7–16.5% in N-use efficiency^32,37^. Other studies also showed that the nutrient supply capacity of biochar in combination with N fertilizer was greater than that of biochar or N fertilizer alone^31,35^. This may have promoted the adsorption function of biochar, which improves N uptake and reduces the N loss^36^. These results indicated that MBCB had the best effect on the N use efficiency, followed by MBC. These two methods are therefore recommended in rice production in Northeast China.

### Effects of different fertilization management practices on soil inorganic N accumulation in the soil profile

Soil inorganic N (SIN) exists mainly as ammonium- and nitrate-N. In the present study, the CRU treatment significantly reduced the contents of NO_3_^−^–N and NH_4_^+^–N and inhibited mineral N accumulation in the soil profile. Previous studies have investigated that CRU could improve nitrogen fertilizer-use efficiency and to reduce the N concentration, N accumulation and residual inorganic N in the soil^19,38^. This may be because CRU controlled the release of nitrogen, reduced the leaching of NO_3_^−^–N and NH_4_^+^–N, and improved nitrogen-use efficiency^39–41^. Moreover, the CRU enhanced N immobilization process could create a temporary reservoir of organic N, which would reduce the potential for SIN leaching in highly leached soils^42,43^. In addition, compared with the MBC treatment, the MBCB treatment significantly reduced the contents of NO_3_^−^–N and NH_4_^+^–N, and had a positive effect on the inhibition of mineral N accumulation in the soil profile. This result was consistent with previous reports^42–44^, and could be attributed to the adsorption and fixation ability of biochar, which could improve soil physical properties, such as the soil CEC and specific surface area, and then increase the contents of NO_3_^−^–N and NH_4_^+^–N in the soil^12,39^. Furthermore, biochar amendment can decrease nitrate leaching and nitrate reduction in the soil, and promote nitrate absorption^45–48^. The effects of CRU and MBCB on the accumulation of SIN became more significant with the increase in the soil profile depth and test year, and the effect on NO_3_^−^–N was greater than that on NH_4_^+^–N, which still needs further investigation.

### Different fertilization management practices affected the balance of N in the soil-plant system

Nitrogen fertilizer application had some effects on the distribution of soil mineral N in the soil profile^48^. In the soil-crop system, the more nitrogen that was uptake by plants, the less N loss or surplus. In the present study, compared with the BU treatment, the CRU treatment significantly reduced the residual SIN, apparent loss of N, and inorganic N surplus. These results were consistent with a previous study, which reported that the nitrate concentration in the 0–130 cm layer of CRU plots was 53% lower than that of BU treated plots at the end of the experiment^49^. This could be attributed to the fact that CRU could improve nutrient uptake and increase nitrogen-use efficiency^41,43^, while excessive utilization of nitrogen fertilizer can reduce plant nitrogen-use efficiency and increase the risk of soil environmental pollution^41^. In addition, lots of residual inorganic N in the BU treatment was mainly due to the low uptake by crops and the poor adsorption and fixation ability of the soil; on the other hand, the small amount of residual inorganic N in the CRU treatment was mainly due to the high uptake by crops and the high utilization rate of nitrogen fertilizer^29^.

In addition, compared with the MBC treatment, the MBCB treatment also reduced the residual SIN, the apparent loss of N, and the inorganic N surplus. This result was in agreement with a previous report^50^, compared with the application of N fertilizer alone, the application of biochar at 20 t ha^−1^ combined with N fertilizer significantly decreased the residual NO_3_^−^-N in the subsoil by 13.2–74.7%^50^. A possible reason was that biochar has a positive effect on the N balance in the soil-crop systems, which could improve crop yield, nitrogen availability, nitrogen-use efficiency and soil physicochemical properties under the MBCB treatment; while the MBC treatment improved only in the rice yield and nitrogen-use efficiency^40,51^.

## Materials and methods

### Experimental site and soil description

A 3-year fixed-site field experiment (2015–2017) was conducted in Fengnian village of Huachuan County, Heilongjiang Province, China. The experimental site is located at 46°40′N and 131°41′E. The climate of the area is characterized as a semi-humid semi-arid cold temperate monsoon climate with an average temperature of 2.5 °C and annual precipitation of 476 mm annually, and a frost-free period of 133 days^52^. The soil type is a Planosol and the soil fertility is relatively low. The soil organic matter content was 18.7 g kg^−1^; the total N, total P and total K contents were 1.63 g kg^−1^, 0.62 g kg^−1^, and 21.6 g kg^−1^, respectively; the available N, available P and available K contents were 113.6 mg kg^−1^, 18.5 mg kg^−1^, and 142.6 mg kg^−1^, respectively; and the soil pH value was 5.37.

### Biochar production and characterization

The biochar used in the field experiment was derived from the pyrolysis of rice straw at 450 °C for 6 h in a vertical kiln, of which approximately 450 kg of biochar and 250 L of wood vinegar were produced per ton of rice straw dry matter. For the field study, the biochar mass was ground to pass through a 2 mm sieve, and then mixed thoroughly to obtain a fine granular consistency that would mix uniformly with the soil mass. The basic physical and chemical properties of the pyrolysis materials and tested biochar were shown in Table 2.

**Table 2:** Essential physical and chemical characteristics of biochar and rice straw.

Materials	Total N (g kg^−1^)	Total P (P_2_O_5_ g kg^−1^)	Total K (K_2_O g kg^−1^)	TOC (C g kg^−1^)	BET surface area (m^2^·g^−1^)	Pore volume (cm^3^·g^−1^)	Pore diameter (mm)	pH
Biochar	8.65	5.7	34.1	487.4	4.88	0.029	16.94	9.86
Straw	14.7	8.9	9.6	386.4				6.73

### Experimental treatments

Five treatments were established for the field experiment. One was no nitrogen fertilizer application; the other four treatments involved the same rates of NPK application, but they were different in terms nitrogen fertilizer management. The treatments were described as follows: (i) CK, no nitrogen fertilizer was applied; (ii) BU, bare urea was applied as basal application; (iii) CRU, controlled-release urea was applied as basal application; (iv) MBC, a mixture of 50% BU and 50% CRU was applied as basal application; and (v) MBCB, a mixture of 50% BU and 50% CRU with 7.5 t ha^−1^ of biochar was applied as basal application. The amounts of nitrogen (N), phosphorus (P_2_O_5_) and potassium fertilizer (K_2_O) were set, and the rates were 187.5 kg ha^−1^, 67.5 kg ha^−1^ and 82.5 kg ha^−1^, respectively, for basic fertilization; 7.5 t ha^−1^ of biochar was applied for the MBCB treatment in the first year of the experiment. The experiment plot area was 24m^2^ (4 m × 6 m) with 3 replications, and the randomized plot arrangement was adopted for the experiment. Bare urea was used as ordinary nitrogen fertilizer (46% N), superphosphate (46% P_2_O_5_) was used as phosphorus fertilizer, potassium chloride (60% K_2_O) was used as potassium fertilizer; and controlled-release urea (CRU, 44% N) and its release longevity was valid for 90 days. The rice variety used for the experiment was Longjing 18, and the transplanting density was 30 cm×15 cm for each hole, with three seedlings in each hole. Three points were selected for each plot, and 1 m^2^ was taken for each point for harvested and grain yield measurement. The yield was then converted (on a dry mass basis) to kilograms per hectare.

### Soil sampling and measurement of inorganic N

The plots were established in accordance with the experimental design. Three initial soil samples from 0–90 cm (0–30, 30–60, 60–90 cm) depth were subsequently collected in the spring of 2015, and other soil samples were collected after harvest in autumn^53^, the same procedures were applied in 2016 and 2017. The soil samples were transported to the lab in cooling boxes and extracted to determine their initial nutrient concentrations with two different methods. The fresh soil samples were extracted for mineral N (NO_3_^–^-N and NH_4_^+^-N) determination by 0.01 mol L^−1^ CaCl_2_ using an auto-analyzer (Model AA3-A001–02E, Bran-Luebbe, Germany). The other soil samples were air dried, ground, passed through a 2-mm sieve, passed through a 0.25-mm sieve, and ultimately stored in paper bags for nutrient analysis.

### Nutrient analysis of plant biomass and nitrogen-use efficiency calculation

The rice plants and grain samples were collected after harvest. In the laboratory, the plant samples were prepared for analysis by first being placed in an oven for 30 min at 105 °C to deactivate enzymes, after which they were dried at 75 °C to a constant weight, weighed, and ground to pass through a 2-mm sieve. The total plant N concentration was determined by H_2_SO_4_-H_2_O_2_ digestion and the micro-Kjeldahl procedure^54^. The N-use efficiency of rice was the difference between the total N uptake of plants in the N application area and the total N uptake of plants in the nitrogen free zone and the percentage of the N application amount (%). The nitrogen agronomic efficiency of the rice was calculated as the ratio of the increase in grain yield relative to the nitrogen application rate (kg kg^−1^). The total N uptake (kg ha^−1^) of the rice was calculated as the sum of the N uptake by the plant straw and the grain yield.

### Data processing and calculation

The statistical software SPSS 21.0 (SPSS Inc.) was adopted for analysis of variance analysis and multiple comparisons based on the LSD method. The SigmaPlot 12.5 software (Systat Inc.) was used to construct the diagrams. The following parameters were calculated by the following formulas:

1 $${\rm{N}}\,{\rm{uptake}}({\rm{kg}}\,{{\rm{ha}}}^{-1})={\rm{grain}}\,{\rm{yield}}\times {\rm{N}}\,{\rm{content}}( \% )+{\rm{straw}}\,{\rm{yield}}\times {\rm{N}}\,{\rm{content}}( \% )$$

Apparent nitrogen-use efficiency (NUE) can also be referred to as apparent nitrogen recovery efficiency. It was calculated as follows:

2 $$NUE( \% )=\frac{{{\rm{N}}}_{f}-{N}_{u}}{{N}_{a}}\times 100$$

where N_f_ is the N uptake (grain plus straw) of the fertilized plot (kg), N_u_ is the N uptake (grain plus straw) of the unfertilized plot (kg) for each replicate, and N_a_ is the quantity of N applied (kg).

Nitrogen agronomic efficiency (NAE), defined as grain production per unit of N applied, was calculated as follows^53,55^:

3 $$NAE(kg\,k{g}^{-1})=\frac{G{Y}_{f}-G{Y}_{u}}{{N}_{a}}$$

where *GY*_f_ is the grain yield of the fertilized plot (kg), *GY*_u_ is the grain yield of the unfertilized plot (kg) for each replicate, and *N*_a_ is the quantity of N applied as nitrogen fertilizer (kg).

The soil inorganic N accumulation was calculated as follows^56,57^:

4 $${{\rm{N}}{\rm{O}}}_{3}^{-}-{\rm{N}}\,{\rm{a}}{\rm{c}}{\rm{c}}{\rm{u}}{\rm{m}}{\rm{u}}{\rm{l}}{\rm{a}}{\rm{t}}{\rm{i}}{\rm{o}}{\rm{n}}({\rm{k}}{\rm{g}}\,{{\rm{h}}{\rm{a}}}^{-1})={\rm{s}}{\rm{o}}{\rm{i}}{\rm{l}}\,{\rm{d}}{\rm{e}}{\rm{p}}{\rm{t}}{\rm{h}}({\rm{c}}{\rm{m}})\times {\rm{s}}{\rm{o}}{\rm{i}}{\rm{l}}\,{\rm{b}}{\rm{u}}{\rm{l}}{\rm{k}}\,{\rm{d}}{\rm{e}}{\rm{n}}{\rm{s}}{\rm{i}}{\rm{t}}{\rm{y}}({\rm{g}}\,{{\rm{c}}{\rm{m}}}^{-3})\times {{\rm{N}}{\rm{O}}}_{3}^{-}-{\rm{N}}\,{\rm{c}}{\rm{o}}{\rm{n}}{\rm{t}}{\rm{e}}{\rm{n}}{\rm{t}}({\rm{m}}{\rm{g}}\,{{\rm{k}}{\rm{g}}}^{-1})/10$$

5 $${{\rm{NH}}}_{4}^{+}-{\rm{N}}\,{\rm{accumulation}}({\rm{kg}}\,{{\rm{ha}}}^{-1})={\rm{soil}}\,{\rm{depth}}({\rm{cm}})\times {\rm{soil}}\,{\rm{bulk}}\,{\rm{density}}({\rm{g}}\,{{\rm{cm}}}^{-3})\times {{\rm{NH}}}_{4}^{+}-{\rm{N}}\,{\rm{content}}({\rm{mg}}\,{{\rm{kg}}}^{-1})/10$$

## Conclusions

Our study demonstrated that the MBCB treatment had a significant effect on rice yield, nitrogen-use efficiency, residual soil inorganic nitrogen and its surplus in soil-plant system, and MBCB had a unique advantage over single application of BU, CRU or MBC. Therefore, to prevent soil degradation caused by the abundant use of chemical fertilizers and to promote high nitrogen-use efficiency, mixed application of biochar and controlled-release nitrogen fertilizer is an effective way to achieve high yield, high fertilization efficiency and sustainable rice production in Northeast China, especially for the local formers.

## Data Availability

The original data can be obtained from the authors upon reasonable request.
